# Effect of ondansetron on QTc interval prolongation in healthy pediatric patients: a systematic review and meta-analysis

**DOI:** 10.1590/1984-0462/2026/44/2025134

**Published:** 2026-03-23

**Authors:** Isabela Maurício Costa Carneiro, Paulo Eduardo Souza Castelo Branco, Adriane Helena Silva Franco, Amanda Prates de Oliveira, Agatha Marcela Andrade de Aguiar

**Affiliations:** a Faculdade de Minas, Belo Horizonte, MG, Brazil.

**Keywords:** Ondansetron, QTc interval, Tp-e interval, Torsades de Pointes, TdP, Ondansetrona, Intervalo QT, Intervalo Tp-e, Torsades de Pointes

## Abstract

**Objective:**

The aim of this study was to describe electrocardiographic changes in healthy pediatric patients receiving low-dose ondansetron and to determine whether these changes are associated with the occurrence of cardiac dysrhythmias.

**Data source:**

The search was conducted in PubMed, EMBASE, LILACS, SciELO, and the Cochrane databases, selecting articles published until September 2024. The primary outcome was the mean change in the corrected QT interval (QTc) interval. The mean variation of the Tp-e interval and the incidence of significant QTc prolongation were assessed as secondary outcomes.

**Data synthesis:**

Four studies were included in this review, including 231 healthy pediatric patients who received ondansetron (IV or oral). Most were male, aged 0.6–18 years. The mean IV ondansetron dose ranged from 0.1 to 0.2 mg/kg, while the mean oral dose was 0.18 mg/kg, with a maximum dose of 8 mg. The mean change in the QTc interval was 4.7 ms (95% confidence interval [CI] 1.48.1), and in the Tp-e interval was 7.7 ms (95%CI 2.0–13.5). The risk of a significant QTc prolongation was 2.5% (95%CI -0.009–0.059). No dysrhythmia was observed in the studies.

**Conclusions:**

There was a statistically significant increase in QTc and Tp-e intervals following ondansetron administration in healthy pediatric patients. However, it is highly unlikely that these changes result in cardiac dysrhythmia, suggesting no relationship between low-dose ondansetron use and an increased risk of dysrhythmia in healthy pediatric patients.

## RESUMO

**Objetivo:**

Descrever as alterações eletrocardiográficas em pacientes pediátricos saudáveis que recebem baixas doses de ondansetrona e determinar sua associação com a ocorrência dearritmias cardíacas.

**Fonte de dados:**

A busca foi realizada nas bases de dados PubMed, Embase, Literatura Latino-Americana e do Caribe em Ciências da Saúde (Lilacs), Scientific Electronic Library Online (SciELO) e Cochrane, selecionando artigos publicados até setembro de 2024. O desfecho primário foi a mudança média no intervalo QTc. A variação média do intervalo Tp-e e a incidência de prolongamento significativo do intervalo QT foram avaliadas como desfechos secundários.

**Síntese dos dados:**

Foram incluídos nesta revisão 4 estudos, envolvendo 231 pacientes pediátricos saudáveis que receberam ondansetrona (IV ou oral) como antiemético. A maioria dos pacientes era do sexo masculino, com idades entre 0,6 e 18 anos. A dose média de ondansetrona IV variou de 0,1 a 0,2 mg/kg, enquanto a dose média oral foi de 0,18 mg/kg, com dose máxima de 8 mg. A mudança média no intervalo QTc foi de 4,7 ms (IC95% 1,4–8,1) e de 7,7 ms (IC95% 2,0–13,5) no intervalo Tp-e após a administração de ondansetrona. O risco de um prolongamento significativo do intervalo QT foi de 2,5% (IC95% -0,009–0,059); nenhuma arritmia foi observada nos estudos.

**Conclusões:**

Houve um aumento significativo nos intervalos QTc e Tp-e após o uso de ondansetrona em pacientes pediátricos saudáveis. Entretanto, é altamente improvável que essas alterações resultem em arritmias ventriculares, demonstrando a ausência de relação entre o uso de dose baixa de ondansetrona e um risco aumentado de arritmia em pacientes pediátricos.

## INTRODUCTION

 Serotonin receptor antagonists, including ondansetron (5-HT3, 5-hydroxytryptamine type 3 receptor), are effective medications for treating nausea and vomiting and are widely used in pediatric patients after surgery, chemotherapy, radiotherapy, or in cases of gastrointestinal tract infections. These medications block the action of serotonin, a substance associated with the development of nausea and vomiting.^1,2^ However, recent studies have shown that ondansetron can induce prolongation of the QT interval, especially at higher doses and in patients with other risk factors for developing cardiac dysrhythmia.^3-5^

 In 2012, the U.S. Food and Drug Administration (FDA) issued a statement withdrawing intravenous ondansetron 32 mg from the market due to prolongation of the QT interval and an increased risk of cardiac dysrhythmia in clinical studies using this dose.^3^ Currently, the FDA recommends the use of low-dose ondansetron (150 mcg/kg) for the treatment of postoperative nausea and vomiting and patients undergoing chemotherapy and radiotherapy, not exceeding the maximum recommended dose of 16 mg.^1,2^

 In an electrocardiogram (ECG), the QT interval is generated by the depolarization and repolarization of the ventricles, measured from the beginning of the QRS complex to the end of the T wave.^6,7^ The corrected QT interval (QTc) is obtained by adjusting for the patient’s heart rate, with Bazett’s formula being the most commonly used method for this correction. Prolongation of the QTc interval may indicate delayed ventricular repolarization or long QT syndrome (LQTS) and is associated with an increased risk of developing the rare, potentially fatal polymorphic ventricular tachycardia known as Torsades de Pointes (TdP).^7^ However, drug-induced QT interval prolongation does not necessarily imply a propensity to cause TdP, as only certain medications appear to be associated with this risk. 

 Although QT interval prolongation is the most commonly used electrical marker for assessing the risk of developing cardiac dysrhythmia, other markers, such as the Tp-e interval, can aid these investigations. The Tp-e interval refers to the duration between the peak and the end of the T wave and suggests the presence of transmural refractory dispersion. This marker can be used for risk stratification in dysrhythmia.^8^ However, it remains challenging to determine which of these markers can reliably predict the occurrence of TdP. 

 Since the FDA announcement, new studies have been evaluating the risk of developing cardiac dysrhythmia in children and adults after the use of lower doses of ondansetron.^5,9,10^ However, in most studies, patients have other risk factors for he development of TdP, such as the use of other drugs that can cause dysrhythmia, cardiac disease, LQTS, and electrolyte disturbances.^11,12^ In this study, we described electrocardiographic changes in healthy pediatric patients who received a low dose of ondansetron for the prevention and treatment of nausea and vomiting and whether the use of this drug increased the occurrence of dysrhythmias in this age group. 

## METHOD

 This systematic review and meta-analysis were registered prospectively at the International Prospective Register of Systematic Reviews (PROSPERO; Registration ID: CRD42024611990). 

 This single-arm meta-analysis included all studies that met our eligibility criteria, based on our PICOT (Population, Intervention, Comparison, Outcome, Type) question; IV (intravenous) question:

1. Randomized controlled trials (RCTs) or non-randomized cohort studies (type of studies),
2. With healthy pediatric patients (population),
3. Who received oral or intravenous ondansetron as an antiemetic (intervention), and
4. Who reported one of the outcomes of interest (outcomes: QT interval, Tp-e interval, TdP).

Studies in which pediatric patients had any risk factor for QT interval prolongation were excluded: heart disease, LQTS, cancer patients, use of other medications that cause QT interval prolongation, and electrolyte alterations. 

 We systematically searched the PubMed, EMBASE, LILACS, SciELO, and Cochrane Central Register of Controlled Trials databases on September 3, 2024, using the following search strategy: (*ondansetron*) AND ((*torsades de pointes*) OR (*QT interval*)) AND (*children*). Two authors independently reviewed the titles and abstracts of all retrieved articles and selected those that answered our PICOT question. A third reviewer resolved disagreements. The references from all included studies and previous reviews were also manually searched. Three authors independently extracted the data after predefined search criteria and quality assessment. A second author confirmed all of the extracted data. 

 The primary outcome of interest is the mean change in QTc interval before and after oral or IV administration of ondansetron. The QT interval is corrected according to Bazett’s formula (QTc=QT/√RR). Our secondary outcomes were (1) the mean change in the Tp-e interval and (2) the incidence of significant QT interval prolongation after ondansetron administration. The Tp-e interval is measured from the peak of the T wave to the end of the T wave. In the presence of a U wave, the end of the T wave is defined as the lowest point between the T wave and the U wave [7]. The Tp-e interval is a noninvasive method that can potentially assess the risk of developing a ventricular dysrhythmia, such as TdP.^7,8^

 We evaluated the risk of bias in randomized studies using the Cochrane Risk of Bias 2 (RoB2) tool.^13^ Non-randomized studies were assessed using the Risk of Bias in Non-randomized Studies — of Intervention tool (ROBINS-I).^14^ Two independent authors completed the risk of bias assessment, and disagreements were resolved by consensus. 

 This systematic review and meta-analysis were performed and reported by the Cochrane Collaboration Handbook for Systematic Reviews of Interventions^15^ and the Preferred Reporting Items for Systematic Reviews and Meta-Analysis (PRISMA) Statement guideline.^16^ Continuous outcomes were compared using mean difference (MD) and standardized mean difference (SMD) with a 95% confidence interval (CI). Heterogeneity was measured using Higgins’ I^2^ statistic. An I^2^>50% was considered significant. The DerSimonian and Laird random effects model was used for all outcomes, as recommended by the Cochrane Collaboration.^15^ For statistical analysis, we used OpenMega[analyst] software (Brown University, United States). 

## RESULTS

**Figure 1:**
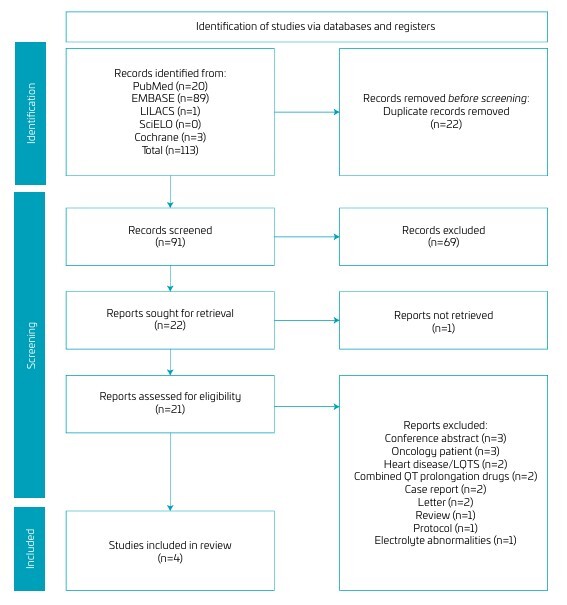
PRISMA flow diagram for study selection.

 Our initial search strategy retrieved 113 articles, 20 from PubMed, 89 from Embase, one from LILACS, and three from Cochrane. No articles were found in the SciELO database. We excluded 22 duplicate articles and screened 91 articles by title and abstract. Of these, 69 articles were excluded because they did not answer our PICOT question, and one could not be retrieved, leaving 21 articles for a full-text review. In the end, only four articles were included in this review, the others being excluded for the reasons reported in Figure 1. 

 This review included 231 healthy pediatric patients who received ondansetron for nausea and vomiting due to acute gastroenteritis or for prophylaxis in patients undergoing elective surgical procedures (one-sided cochlear implantation, bilateral strabismus, otoplasty, or dental surgery). Two studies were randomized controlled trials conducted in Canada and Iran, while the others were retrospective observational studies conducted in the United States and the Republic of Korea (Table 1)^7,8,17,18^. Most patients were male, aged 0.6–18 years. One hundred fifty-one patients received intravenous ondansetron at 0.1–0.2 mg/kg. In contrast, the patients in the study by Yang et al.^17^ eceived an average oral ondansetron dose of 0.18 mg/kg, with the maximum dose across all studies and both routes of administration being 8 mg (Table 2)^7,8,17,18^. The outcomes evaluated are the mean variation in the QTc and Tp-e intervals after ondansetron administration and the incidence of significant QT prolongation in the population studied. 

**Table 1:** Baseline characteristics of included studies.

Study	Population	Intervention	Country	Control group	Number of patients (n)		Age (SD)	
					Intervention	Control	Intervention	Control
Mehta et al.^7^	Healthy children for elective bilateral strabismus, otoplasty or dental surgery	IV Ondansetron	Canada	Saline	20	20	4.9 yrs (1.9)	4.8 yrs (1.7)
Krammes et al.^18^	Pediatric patients in the ED who receive IV ondansetron for vomiting, nausea, or inability to take oral fluids	IV Ondansetron	US	*	100	*	4.3 yrs (4.74)	*
Safaeian et al.^8^	Pediatric patients for elective one-sided cochlear implantation	IV Ondansetron	Iran	Dexamethasone	31	30	38 mo (NR)	40 mo (NR)
Yang et al.^17^	Mildly to moderately dehydrated children with AGE	Oral Ondansetron	Republic of Korea	*	80	*	53.3 mo (32.4)	*

n: number; SD: standard deviation; IV: intravenous; yrs: years; ED: emergency department; mo: months; NR: not reported; AGE: acute gastroenteritis.

* Not applicable, as this is a retrospective observational study without a control group.

**Table 2:** Ondansetron dose and outcomes of included studies.

Study	Intervention	Weight, Kg (SD)	Dose of ondansetron	ECG time	Measured by	Outcomes
Mehta et al.^7^	Intravenous Ondansetron	18.8 (5.2)	100 mcg/kg	Preoperative+5 min after ondansetron	Pediatric cardiologist	QTc interval Tp-e interval QT Prolongation
Krammes et al.^18^	Intravenous Ondansetron	34.7 (21.68)	150 mcg/kg	ECG baseline+3 min after ondansetron	Cardiologist	QTc interval QT Prolongation
Safaeian et al.^8^	Intravenous Ondansetron	NR	200 mcg/kg	Preoperative+15 min after ondansetron	Cardiologist	QTc interval Tp-e interval QT Prolongation
Yang et al.^17^	Oral Ondansetron	18.9 (10.0)	180±40 mcg/kg	ECG baseline+65min after ondansetron	Emergency physician/Cardiologist	QTc intervalQT Prolongation

SD: standard deviation; ECG: electrocardiogram; NR: not reported.

**Figure 2:**
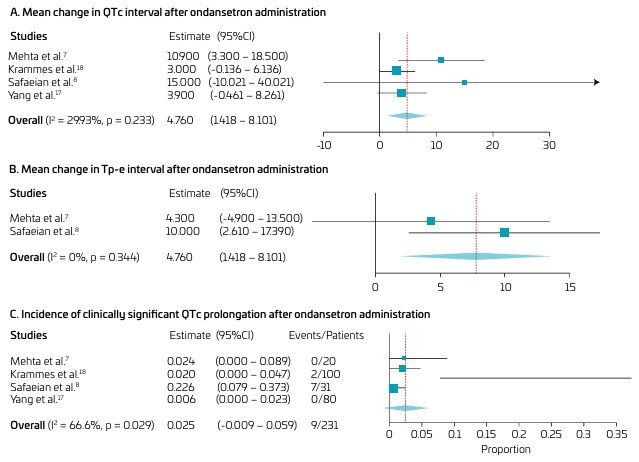
Forest plot of the outcomes of this review. A: Study weights: Mehta et al.^7^: 15.695%; Krammes et al.^18^: 48.096%; Safaeian et al.^8^: 1.746%; Yang et al.^17^: 34.463%. Model Results: Estimate 4.760; Lower bound 1.418; Upper bound 8.101; Std. error 1.705; p-value 0.005. Heterogeneity: I^2^: 29.93%. B: Study weights: Mehta et al.^7^: 39.218%; Safaeian et al.^8^: 60.782%. Model Results: Estimate 7.764; Lower bound 2.003; Upper bound 13.526; Std. error 2.940; p-value 0.008. Heterogeneity: I^2^: 0%. C: Study weights: Mehta et al.^7^: 17.459%; Krammes et al.^18^: 35.905%; Safaeian et al.^8^: 4.909%; Yang et al.^17^: 41.727%. Model Results: Estimate 0.025; Lower bound -0.009; Upper bound 0.059; Std. error 0.018; p-value 0.155. Heterogeneity: I^2^: 66.66%.

 Healthy pediatric patients receiving ondansetron for the treatment and prevention of nausea and vomiting had a mean change in QTc interval from baseline to post-drug administration of 4.7 ms (95% CI 1.4–8.1; p=0.005; I^2^=30%; Figure 2A). Sensitivity analysis was performed to assess the effect of each trial on the synthesis. Exclusion of the Krammes et al.^18^ and Mehta et al.^7^ studies showed the largest and smallest variation in the QTc interval, respectively, ΔQTc=6.8 ms vs. ΔQTc=3.4 ms. Moderate overall heterogeneity was reported when the Yang et al.^17^ study was excluded from the sensitivity analysis (I^2^=53%). 

 The mean change in Tp-e interval after intravenous ondansetron administration was 7.7 ms (95%CI 2.0–13.5; p=0.008; I^2^=0%, Figure 2B). The incidence of clinically significant QTc prolongation, defined as QTc >500 ms or ΔQTc >60 ms, was 2.5% (95%CI -0.009–0.059; p=0.155; I2=67%, Figure 2C).^19^ Moderate overall heterogeneity was reported in this analysis. No cardiac dysrhythmia or TdP episodes were reported. 

 The risk of bias from randomized clinical trials is reported in Table 3 ^7,8^, in which all studies obtained a low risk of bias in the five domains assessed by RoB2,^13^ presenting a low overall risk of bias. Retrospective observational studies had severe risks of bias in the confounding domain, as the authors did not clarify in the exclusion criteria whether they excluded all confounders that could prolong the QT interval, such as electrolyte alterations in pediatric patients. For this reason, Krammes et al.^18^ and Yang et al.^17^ obtained an overall severe risk of bias (Table 4)^17,18^. 

**Table 3:** Risk of bias summary from randomized clinical trials.

Study	Risk of bias domains					
	D1	D2	D3	D4	D5	Overall
Mehta et al.^7^	Low	Low	Low	Low	Low	Low
Safaeian et al.^8^	Low	Low	Low	Low	Low	Low

Domains: D1: bias arising from the randomization process; D2: bias due to deviations from intended intervention; D3: bias due to missing outcome data; D4: bias in measurement of the outcome; D5: bias in selection of the reported result.

**Table 4:** Risk of bias summary from non-randomized clinical trials.

Study	Risk of bias domains							
	D1	D2	D3	D4	D5	D6	D7	Overall
Krammes et al.^18^	Serious	Low	Low	Low	Low	Low	Low	Serious
Yang et al.^17^	Serious	Low	Low	Low	Low	Low	Low	Serious

Domains: D1: bias due to confounding; D2: bias due to selection of participants; D3: bias in classification of interventions; D4: bias due to deviations from intended interventions; D5: bias due to missing data; D6: bias in measurement of outcomes; D7: bias in selection of the reported result.

## DISCUSSION

 In this systematic review and single-arm meta-analysis, we included four studies that evaluated the mean change in the QTc interval after the administration of ondansetron as an antiemetic, showing a statistically significant increase of 4.7 ms above the baseline ECG (95%CI 1.4–8.1; p=0.005), although the change was not clinically significant enough to cause a worrying prolongation in the QTc interval. An increase in the QT interval of 60 ms or more is already considered a significant concern for the emergence of TdP, with the recommendation to discontinue the drug and to monitor the patient rigorously.^19^

 Analyzing each included study, we can observe that the Safaeian et al.^8^ trial showed a greater mean QTc interval variation (15 ms) and a higher incidence (22.6%) of significant QT prolongation after ondansetron administration. This can be explained by the patients receiving a higher dose of the drug (200 mcg/kg) and being younger pediatric patients (mean: 40 months), compared to the other studies. This result confirms the same findings of the randomized clinical study used by the FDA to suspend higher doses of ondansetron in the United States.^3^

 The FDA study showed that healthy adults who received high doses of IV ondansetron had a more significant prolongation of the QT interval compared to those who received lower doses: 32 mg ΔQTc=19.5 ms; 24 mg ΔQTc=14 ms; 16 mg ΔQTc=9.1 ms; 8 mg ΔQTc=5.6 ms, respectively.^20,21^ The Rukerd et al.^4^ study showed that the incidence of significant QTc prolongation was higher in patients receiving 8 mg of ondansetron than in those receiving lower doses. However, another observational study conducted in Turkey did not show a statistically significant variation between different doses of ondansetron.^22^

 Another factor that may explain the high incidence of QT prolongation observed in Safaeian et al.^8^ trial is that some patients may have Jervell and Lange-Nielsen syndrome, have not been diagnosed, and/or have not presented with QT interval prolongation until the time of cochlear implant surgery. Jervell and Lange-Nielsen syndrome is an autosomal recessive disease in which patients present with sensory-neural hearing loss (SNHL) associated with congenital cardiovascular heart disease, in which these patients generally present with significant QT interval prolongation and an increased risk of ventricular tachycardia, such as TdP.^23^ Another study, also conducted in Iran, revealed that 12.3% of pediatric patients (mean age of 42.6 months) with SNHL who underwent cochlear implants presented significant QT interval prolongation, demonstrating a higher prevalence in this population than in the healthy pediatric population without SNHL, which is explained by the possible presence of Jervell and Lange-Nielsen syndrome in this population.^23^ In this study, similar to that observed in Safaeian et al.^8^ trial, no patient presented any arrhythmia or cardiac complication in the perioperative or postoperative period. 

 Compared with other studies of healthy adult patients who received low doses of ondansetron (4 mg), we observed that the variation in the QT interval in healthy pediatric patients was much smaller than that in adults. While in our study the QT interval variation was 4.7 ms, in other prospective studies of healthy adults, the average QTc prolongation after ondansetron administration ranged from 8.5 to 20 ms.^22,24,25^ This disparity in the data indicates that sensitivity to the effect of ondansetron on the QT interval may be age-dependent, raising the hypothesis that the pharmacological mechanisms or the cardiac tissue response to the drug differ between children and healthy adults. 

 The incidence of significant QT prolongation after ondansetron use, defined in our study as a QTc interval >500 ms or a QTc interval variation >60 ms, was only 2.5% and was not statistically significant.^26^ Although ondansetron use caused QT prolongation, no severe arrhythmic events, such as TdP, were documented. These results are similar to those of studies in adult patients, in which no ventricular dysrhythmia episodes occurred.^4,20,21^ This shows that the induction of significant QT interval prolongation by drugs is not synonymous with the drug having the property of causing ventricular dysrhythmia in healthy children.^7^ However, it is important to emphasize that QT prolongation establishes a substrate that can predispose individuals to dysrhythmia, even if not all patients with this finding will develop them. Consequently, children who exhibit this effect, particularly those with other predisposing factors, require enhanced medical vigilance.^27^ Healthcare professionals must be aware of the risk in patients with specific risk factors, such as baseline QTc prolongation, electrolyte disturbances, concomitant use of other QT-prolonging drugs, and younger pediatric patients with a low body mass index, as this subgroup is more likely to experience significant QTc variation compared to other healthy children.^17,18^

 Our study showed that the mean change in the Tp-e interval after intravenous ondansetron administration in healthy children was 7.7 ms, a larger change than the change in the QT interval of the same patients (ΔQTc=4.7 ms). Although some studies have shown that prolongation of the Tp-e interval is associated with a higher risk of dysrhythmia and sudden death, especially when associated with QT prolongation, our analysis does not support this theory, as no cardiac arrhythmias were observed despite this electrophysiological change.^28,29^ Furthermore, there are still doubts about its applicability, measurement, values, and variability factors related to the Tp-e interval. Therefore, it should not be analyzed in isolation to assess dysrhythmia risk, and it is essential to evaluate other ECG markers, such as QT interval prolongation. 

 The Yang et al.^17^ study was the only one to administer oral ondansetron and, consequently, the only one to perform ECG assessments 1 h after administration, aligning with the peak plasma concentration time of 30–120 min for the oral formulation.^30^ This methodology contrasts with the other included studies, which utilized intravenous administration and obtained ECGs much earlier, between 3 and 15 min post-dose. This earlier timing is supported by previous pharmacokinetic and pharmacodynamic studies, which demonstrate that the maximum QT interval prolongation following intravenous ondansetron occurs between 3 and 5 min and persists for at least 15 min.^22,25^

 Our study has some limitations. First, randomized clinical trials and observational studies were included, with nonRCTs subject to unreported risks of bias, such as observational studies not reporting whether all risk factors for TdP were excluded from the selected population (e.g., electrolyte disturbances). Second, the studies included in this meta-analysis are subject to type II error (β) due to the small sample sizes, and the findings of this analysis may not reflect all healthy pediatric patients. A further limitation is the substantial heterogeneity observed in the analysis of significant QT prolongation. This heterogeneity originated primarily from a marked discrepancy between the prevalence reported in the Safaeian et al.^8^ trial and the other studies. As previously discussed and demonstrated in other research, the incidence of QT prolongation is higher in patients with sensory-neural hearing loss. Consequently, the pooled prevalence from our meta-analysis may be biased and may not accurately represent the true prevalence in the general healthy pediatric population without congenital hearing loss. Finally, the lack of standardization in the route of administration and the dosage of ondansetron across the studies is another limitation, especially given that the studied outcomes are known to be dose-dependent. However, it is important to note that most of the included studies utilized a low dose, as recommended by the U.S. Food and Drug Administration.^1,3^

 Ondansetron use in pediatric patients should be individualized and cautious, considering concomitant risk factors such as baseline QT prolongation, electrolyte disturbances, or the associated use of other medications that prolong the QT interval, which may increase the risk of ventricular dysrhythmia. Further randomized clinical trials with larger sample sizes are needed to clarify the clinical significance of the altered Tp-e interval and to better elucidate the risk of developing cardiac dysrhythmia, such as TdP. 

## CONCLUSIONS

 In conclusion, ondansetron administration in healthy children is associated with statistically significant increases in both the QTc and Tp-e intervals. However, these alterations did not translate into a clinically observable risk of ventricular dysrhythmia, supporting the safety of low-dose ondansetron (≤8 mg or 150 mcg/kg) in a controlled setting. 

## Data Availability

The database that originated the article is available with the corresponding author.
